# Parent and household influences on calcium intake among early adolescents

**DOI:** 10.1186/s12889-018-6297-5

**Published:** 2018-12-19

**Authors:** Jinan Banna, Jessica O’Driscoll, Carol J. Boushey, Garry Auld, Beth Olson, Mary Cluskey, Miriam Edlefsen Ballejos, Christine Bruhn, Scottie Misner, Marla Reicks, Siew Sun Wong, Sahar Zaghloul

**Affiliations:** 1 Department of Human Nutrition, Food and Animal Sciences, College of Tropical Agriculture and Human Resources, University of Hawai‘i at Mānoa, Agricultural Sciences 216, 1955 East-West Rd, Honolulu, HI 96822 USA; 2 Department of Nutrition and Dietetics, Tallaght University Hospital, Dublin, Ireland; 3 Purdue University, 700 W State St. West Lafayette, Lafayette, IN 47907 USA; 4 Epidemiology Program, University of Hawaii Cancer Center, 701 Ilalo Street, Room 525, Honolulu, Hawaii 96813 USA; 5 Department of Food Science & Human Nutrition, Colorado State University, 210 105 Gifford Building, Fort Collins, CO 80523-1571 USA; 6 Department of Nutritional Sciences, University of Wisconsin-Madison, Madison, WI 53706 USA; 7 Food Science & Human Nutrition, Michigan State University, East Lansing, USA; 8 Nutrition, School of Biological and Population Health Sciences, Oregon State University, 200 Milam Hall, Corvallis, OR 97331-5103 USA; 9 Puyallup Research and Extension Center, Washington State University, 2606 W Pioneer Way, Puyallup, WA 98371-4998 USA; 10 Davis, Food Science and Technology, University of California, One Shields Road, Davis, CA 95616-8598 USA; 11 Department of Nutritional Sciences, University of Arizona, 309 Shantz, Tucson, AZ 85721-0038 USA; 12 Department of Food Science and Nutrition, University of Minnesota, 1334 Eckles Ave, St. Paul, MN 55108 USA; 13 College of Public Health and Human Sciences, 105H Ballard Hall, Corvallis, OR 97331 USA; 14 Old Main Hill, Utah State University, Logan, UT 84322 USA; 15 National Nutrition Institute, 16 Kasr El Aini Street, Cairo, Egypt; 16 University of Hawaii, Ag Sci, 1955 East-West Road, Honolulu, HI 96822 USA

**Keywords:** Calcium, Parents, Early adolescent children, Calcium-rich foods, Cross-sectional, Dairy, Asian, Hispanic, Non-Hispanic white

## Abstract

**Background:**

Calcium intake during early adolescence falls short of requirements for maximum bone accretion. Parents and the home food environment potentially influence children’s calcium intakes. This study aimed to quantify parental psychosocial factors (PSF) predicting calcium intakes of Asian, Hispanic, and non-Hispanic white (NHW) early adolescent children from a parental perspective.

**Methods:**

This was a cross-sectional study involving the administration of a validated calcium-specific food frequency questionnaire to a convenience sample of children aged 10–13 years and the primary individual responsible for food acquisition in the child’s household. Based on Social Cognitive Theory, parental factors potentially associated with children’s calcium intake were also assessed via parent questionnaires. The total study sample consisted of 633 parent-child pairs (Asian = 110, Hispanic = 239, NHW = 284). Questionnaires were completed at community-based centers/sites. Outcome measures were the association between parent-child calcium (mg), milk (cups/day), and soda (cans/day) intakes and the predictive value of significant parental PSF towards calcium intakes of their children*.* Sex-adjusted linear regression and multivariate analyses were performed.

**Results:**

Calcium intakes of parent-child pairs were positively associated among all ethnic groups (*r* = 0.296; *P* < 0.001). Soda intakes were positively associated among Hispanic parent-child pairs only (*r* = 0.343; *P* < 0.001). Home availability of calcium-rich foods (CRF), parental rules and expectations for their child’s intake of beverages, and parents’ calcium intake/role modeling were positively associated with children’s calcium intake and overwhelmed all other PSF in multivariate analyses. Significant cultural differences were observed. Parental role modeling was a significant factor among Hispanic dyads only. Multivariate models explained 19–21% of the variance in children’s calcium intakes.

**Conclusions:**

Nutrition interventions to improve children’s calcium intakes should focus on parents and provide guidance on improving home availability of CRF and increasing rules and expectations for the consumption of CRF. Among Hispanic families, interventions promoting parental modeling of desired dietary behaviors may be most successful.

**Electronic supplementary material:**

The online version of this article (10.1186/s12889-018-6297-5) contains supplementary material, which is available to authorized users.

## Background

An adequate calcium intake throughout adolescence reduces the risk of osteoporosis in later life through greater bone acquisition during growth [1]. The achievement of the recommended intake for calcium depends largely upon the inclusion of dairy products in the diet [2]. However, previous findings indicate that during this important period of bone accretion, adolescents have inadequate intakes of dairy products [3] and calcium [4, 5]. Several studies have reported lower calcium intakes among Asian [6, 7] and Hispanic [6] adolescents compared to other ethnic groups. The public health significance of this disparity is magnified by evidence suggesting an increased risk for osteoporosis among Asian and Hispanic populations [8]. Therefore, nutrition interventions which aim to improve calcium intakes of adolescents are urgently required. In order to design effective intervention programs, an understanding of factors influencing calcium intakes of specific target groups is required.

Parents have been identified as an essential component of the social environment influencing their children’s dietary choices [9]. The increasing independence experienced during the transition from childhood to adolescence [10] and the greater influence from peers and the media [11] would suggest that parental influence on their children’s calcium intakes declines during the early adolescent years. On the other hand, studies show parents may positively influence their early adolescent children’s calcium intakes through behaviors such as role modelling [12, 13], controlling the availability of calcium-rich foods (CRF) in the home [13–15], preparing and sharing family meals [14, 16], educating children about the importance of calcium for bone health [15], and encouraging the consumption of CRF [15].

The majority of previous studies examining parental influences on children’s calcium intakes have not included cross-cultural comparisons [17–19]. Findings that calcium intakes and risk for osteoporosis are not uniform among all ethnic groups underscore the need to include cross-cultural comparisons in further investigations in this research area. Studies involving various ethnic groups have focused on parental influences from the child’s perspective only [6, 14, 17, 20, 21] or have adopted a qualitative approach [15, 22, 23]. Therefore, there is currently a lack of quantitative data on the association between the home food environment and calcium intakes from the parents’ perspective of their early adolescent children. Developing effective family-based intervention programs requires a thorough understanding of parents’ perceptions of their role in influencing their children’s dietary choices [24].

**Fig. 1:**
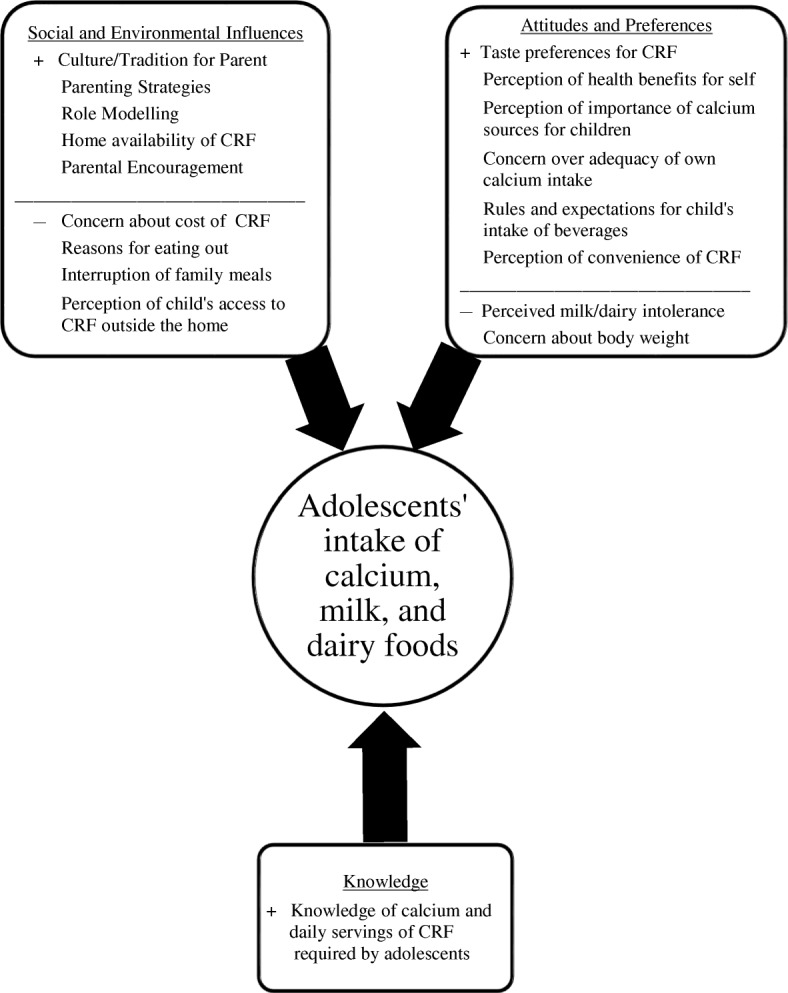
Conceptual model based on Social Cognitive Theory of parental psychosocial factors influencing their early adolescent children’s intake of calcium, milk and other calcium-rich foods (CRF)

While qualitative research has suggested an association between specific parenting practices and calcium intakes of their children [15], no efforts have been made to identify the most salient parental psychosocial factors (PSF) in this context. As demonstrated in Fig. 1, Social Cognitive Theory (SCT) [25] would indicate that there are multiple parental PSF potentially influencing children’s calcium intakes. The most salient parental factors associated with children’s calcium intakes need to be identified [26–28]. As noted by Blanchette and Brug [29], “interventions aiming to improve health-related behaviors should be tailored to the most important determinants or mediators of these behaviors in order to be successful.” Therefore, the aim of the current study was to identify the most salient parental PSF (social and environmental factors, attitudes and preferences, and knowledge) associated with calcium intakes of their early adolescent children, using cross-cultural comparisons and parent-child dyads, in order to quantify the variance in children’s calcium intakes explained by these factors. Research hypotheses included 1) parental psychosocial factors and household factors will be significantly associated at the *P* < 0.05 level with calcium intakes of their early adolescent children (See Fig. 1 for hypothesized directions of these associations), and 2) estimated calcium intakes of parents responsible for household food acquisition and preparation and the estimated calcium intakes of their early adolescent children aged 10–13 years will be significantly associated at the *P* < 0.05 level, as measured by the same food frequency questionnaire.

## Methods

### Study design and sample

This study was part of a United States Department of Agriculture (USDA) multi-state project involving institutions from 11 states in the U.S: Arizona (AZ), California (CA), Colorado (CO), Hawaii (HI), Indiana (IN), Kentucky (KY), Michigan (MI), Minnesota (MN), Oregon (OR), Utah (UT), and Washington (WA). The study used a cross-sectional design and a purposive sampling scheme to recruit children aged 10–13 years old and the primary person responsible for food acquisition and preparation in the child’s household. Results from the National Osteoporosis Risk Assessment [7] identified Asian, Hispanic, and non-Hispanic white (NHW) women as being at highest risk for osteoporosis; therefore, these ethnic groups were targeted for this study. Other inclusion criteria for both parents and their children were: 1) the ability to read and speak English and 2) having lived in the U.S for at least 12 months.

The demographic profiles of 9 of the states, i.e., AZ, CA, CO, HI, MI, MN, OR, UT and WA were used to recruit approximately equal proportions of parent-child dyads from each ethnic group. Recruitment techniques designated as appropriate for this study were fliers, verbal announcements, personal contacts, written announcements in bulletins and newsletters, and presentations to groups. In order to ensure diversity of the study sample in terms of level of education and socio-economic status (SES), a wide range of organizations and groups were approached for this study. These included the Expanded Food and Nutrition Education Program (EFNEP), Supplemental Nutrition Assistance Program Education (SNAP-Ed/formerly Food Stamp Nutrition Education Program), faith-based groups, after-school programs, scouting groups, non-elite competitive age-group sports teams, and the Special Supplemental Nutrition Program for Women, Infants, and Children (WIC).

Data were collected between April 2006 and September 2008. A standardized data collection protocol was developed and used to administer the parent questionnaires and the child questionnaires in a consistent manner across all sites. The questionnaires were designed to be independently self-administered by parents and their children. Data were collected in community settings, such as community centers, libraries, and athletic facilities, and respondents’ homes. Researchers in several states also asked parents to return completed questionnaires with a stamped return envelope after obtaining consent in person (14–50% of questionnaires for these states). Parents took approximately 20–45 min, and children approximately 20–30 min to complete the questionnaires. All questionnaires were completed in English. Based on each research institution’s appropriate level of remuneration, parents and children were each given $5–$20 in cash or as gift certificates in return for their participation. All phases of this study were approved by the Institutional Review Board of each respective study site.

A previous study [18] reported a significant correlation in frequency of milk consumption between mother-child dyads based on a study sample of 180 pairs. Thus, it was assumed that a similar sample size was required for the current study in order to detect a significant association between parental psychosocial factors and their children’s calcium intakes. A total of 673 parent questionnaires and 680 child questionnaires were sent to a central location for scanning. In some instances, the adult respondent was a relative or caregiver other than the parent. For simplicity, the adult respondent is referred to as “parent” in this report.

### Questionnaires and variables assessed

#### Parent and child characteristics

The adult questionnaire included questions regarding age, sex, education, and employment status of both the participant and his/her spouse. In addition, information on household characteristics, such as the number of adults and children living in the home, age and sex of the adolescent participant and his/her siblings, and participation in public assistance programs was collected. The child questionnaire requested information on age, sex, grade, and family members living in the home. Both the parent and child, in their respective questionnaires, were asked to select their ethnicity (Hispanic/Latino or Not Hispanic/Latino) followed by the race groups with which he/she most identified, as directed by the U.S Office of Management and Budget [30].

#### Dietary assessment

Dietary calcium intakes of early adolescents were estimated (mg/day) through the use of a 79-item calcium-specific semi-quantitative food frequency questionnaire (FFQ), which was previously developed and evaluated for use with Asian, Hispanic, and NHW adolescents [31]. The FFQ asked participants to recall food and beverage consumption of the past month. Further details on the development of the FFQ have been published elsewhere [31]. Although developed for multiethnic youth, this questionnaire has been evaluated for use with adult women [32]. Therefore, the same FFQ was used with parents to allow for direct comparison of calcium intakes (mg) between parents and their children. The questionnaire also included questions regarding eating patterns and current supplement use.

Estimated calcium intakes (mg) were calculated following the procedures described previously [31]. Estimated daily calcium intakes < 100 mg or > 2500 mg were considered implausible [33] and individuals with such values were excluded from analysis involving calcium intakes. Fifteen parents (2%) and 25 children (4%) had implausible calcium intakes. Therefore, 618 parents and 608 children representing 597 parent/child pairs were retained for statistical analyses of calcium intakes. All parents with implausible calcium intakes were Hispanic. Among children, 12 were Hispanic, 11 NHW, and 2 Asian (chi-square = 2.05, df = 2, *P* = 0.360). Dietary calcium intakes (mg) were classified into 2 categories previously used [33]: Total dairy calcium (mg) represented the sum of calcium from dairy foods and calcium from mixed foods. An example of a mixed food containing both dairy and non-dairy calcium sources is pizza where calcium from the cheese is from a dairy source plus a non-dairy calcium source from the crust. Total calcium from food (mg) was the sum of total dairy calcium, calcium from non-dairy foods (e.g., broccoli, calcium-fortified orange juice), and mixed foods. Both calcium intakes were summarized as mg/day. Fluid milk consumption was defined using the food grouping, “milk to drink, white or chocolate.” The frequency responses were recoded to cups of milk per day (e.g., 1–3 cups per month would be 0.0667 cup/day; 1 cup per week would be 0.1429 cups per day and so forth). Similarly, soda pop consumption was re-coded to cans per day.

#### Parental psychosocial factors

Based on SCT [25] and findings from previous studies [15, 22, 23], a questionnaire was developed to assess parental PSF hypothesized to influence children’s calcium intakes (Additional file 1). Three major constructs were defined: 1) social and environmental factors, 2) parent’s attitudes and preferences, and 3) parental knowledge of calcium intake during adolescence. These constructs were measured with 17 subscales. Briefly, for the social and environmental construct, 9 subscales emerged: culture/tradition for parent (calcium rich foods parent grew up with or considered culturally relevant), parenting styles (require or encourage child to eat food), parental role modeling of intake of CRF (CRF served at eating occasions throughout the day, as well as soda), parental concern about cost of CRF, availability of CRF in the home, parental encouragement of healthy eating practices (including talking to the child about eating healthfully), reasons for eating out, interruption of family meals, and parental perception of child’s access to CRF outside the home. Eight subscales were identified for the attitudes and preferences construct: parental taste preferences for CRF, parent’s perceived intolerance to milk and dairy foods, parental perception of health benefits of CRF for self, parental perception of the importance of calcium food sources for children, parental concern about dairy foods negatively influencing body weight, parental concern over adequacy of own calcium intake, parental expectations for child’s intake of beverages, and parental perception of convenience of CRF. In a separate study, these subscales met standards for psychometric properties with modest to acceptable Pearson correlation test-retest reliability coefficients for some subscales (0.68–0.85) and Cronbach α coefficients for internal consistency (0.50–0.79) [33, 34]. The scale composition, scale items and response categories have been published previously [35].

The raw data responses to the statements and questions representing each individual subscale were summed and then an average response was calculated for each subscale. In general, each response was coded as 1 to 5 or 1 to 6. The final score for a subscale of 5 statements would be the sum of the responses to the 5 statements divided by 5. As appropriate, the values associated with some responses were reverse coded for accurate directionality. For subscales containing 3 or more statements, a response was needed for at least 66% of the statements to calculate a score.

#### Data analysis

All questionnaires were reviewed and corrected for stray marks and multiple responses prior to sending to a central location for optical scanner reading. Scanning was completed in batches and separate raw data files were sent to a second central location for data cleaning and preparation for analysis. Of the parent questionnaires scanned, 4 were blank, 1 had a data scanning error, and 27 self-reported information outside of the inclusion criteria (i.e. missing ethnicity; ethnicity other than Asian, Hispanic, or NHW; or child’s age) resulting in a total of 641 acceptable questionnaires. Of the scanned child questionnaires, 5 were blank, 2 had data scanning errors, and 5 self-reported ages outside of the inclusion criterion resulting in 668 acceptable questionnaires. Only paired children and parents were retained for statistical analysis (*n* = 633). A flow diagram showing the details of the final sample size is provided in Additional file 2.

When discrepancies existed between the parent and the child responses regarding age, sex, and ethnicity of the child, the parent response was considered accurate and used in all analyses. Participants who selected multiple ethnic groups were classified as Asian, Hispanic, or NHW if one of these groups was also selected. Those who chose Asian and NHW, or Hispanic and NHW, were classified as Asian or Hispanic, respectively. In instances where Asian and Hispanic were both selected (*n* = 3), the predominant ethnic group of the sampling location was used for classification as either Asian or Hispanic.

Data were assessed for normality using normal probability plots and no variable was found to need a transformation. Season was defined as winter (November to January), spring (February to April), summer (May to July), and fall (August to October); employment status of parent was collapsed into no formal employment (student, homemaker/househusband, not employed and retired as they appeared on the questionnaire), part-time employment, and full-time employment; employment status of the household was defined as no formal employment and at least half-time employment of one individual; education level of parent was defined as high school diploma or less, some college, and 4-year college or more; parents’ ages were collapsed into 18–40 years and 41+ years; number of adults living in the home was defined as one, two, and three or more; and number of children in the household was collapsed to one, and two or more. Participation in federally funded programs was positive if the parent reported participation in WIC, SNAP, and/or free/reduced priced school lunch. The child’s report of breakfast location was collapsed to at home, at school, skips breakfast, and other (i.e. fast food restaurant, convenience store, other).

Analysis of variance was used to identify differences in quantitative variables among ethnic groups. Post-hoc Bonferroni analyses were performed on significant *P*-value results to identify which ethnic groups were significantly different. Differences between categorical variables were assessed using chi-squared analysis. Pearson’s correlations and linear regression accounting for sex were completed to examine the relationship between parent-child calcium intakes.

Multivariate linear regression analysis was conducted to quantify the predictive value of significant parental PSF towards calcium intakes (mg) of their early adolescent children while accounting for ethnicity. Parental psychosocial factors and parental calcium intake (mg) were modeled as predictors and children’s total calcium intake (mg) from food was the dependent variable. Potential confounding variables examined were age (of child and parent), soda intakes (of child and parent), and the indicator variables of season, sex (of child and parent), employment status (of parent and household), education level, number of adults in home, number of children in home, single household (yes/no), and participation in federally funded programs. Only those confounding variables significantly altering the association between parental PSF and children’s calcium intakes (mg) were retained in the models. Interaction terms were then examined and only significant interaction terms were retained in the models. Since parental role modeling and parent’s calcium intake (mg) were highly correlated (*r* = 0.491, *P* < 0.001) these were interchanged in separate models. The same statistical models were used with total dairy calcium (mg) as the dependent variable. To account for the complex sampling design, indicator variables for the states were examined with all final models. The results did not differ. All analyses were performed using Statistical Package for the Social Sciences (SPSS) software 17.0 for Windows (2007, Chicago, IL, USA). *P* ≤ 0.05 was used as a measure of statistical significance in all analyses.

## Results

### Characteristics of study sample

Self-reported information indicated the ethnic composition of the study sample was 17% Asian, 38% Hispanic, and 45% NHW. The majority of adult respondents were parents (97%); however, 7 were grandparents, 3 were siblings, and 2 were aunts. Approximately 80% of participants reported living in the U.S for more than 10 years, while only 2% reported living in the U.S for less than 4 years.

Selected characteristics and eating behaviors of children and selected characteristics of their parents stratified by ethnic group are presented in Table 1. Children were equally distributed by sex but not by age, within each ethnic group. Statistically significant differences in dietary behaviors were seen between ethnic groups. Hispanic children were more likely to report participation in the school breakfast program and less likely to report use of supplements containing calcium compared to other ethnic groups. The proportions for the youngest age group, participation in federally funded nutrition programs, 3 or more children in the household, high school education or less, and no formal employment were higher among the Hispanic parents compared to the Asian and NHW parents. Conversely, the Hispanic parents were less likely to report the use of calcium-containing supplements compared to the other ethnic groups.

**Table 1:**
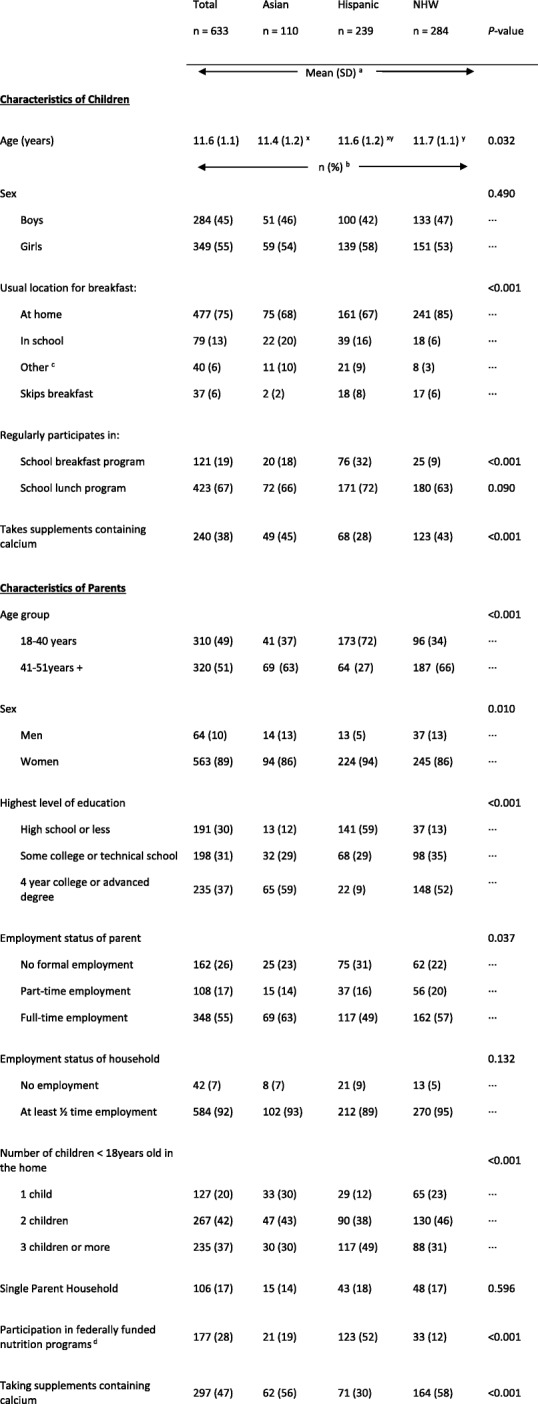
Selected characteristics and eating behaviors of Asian, Hispanic and non-Hispanic white early adolescent children aged 10–13 years and selected characteristics of their parents

*NHW* non-Hispanic white, *SD* standard deviation

^a^Values with different superscripts (x, y) indicate significantly different means between ethnic groups

^b^Numbers do not always add up to 100% due to rounding and missing data

^c^‘Other’ includes fast-food restaurant, convenience store, other, or not specified

^d^Federally funded programs included: Special Supplemental Nutrition Program for Women, Infants, and Children, Supplemental Nutrition Assistance Program, and free/reduced price school lunch

### Calcium intakes of study sample

Estimated mean daily calcium (mg), milk (cups/day), and soda (cans/day) intakes of Asian, Hispanic, and NHW parents and their children as estimated from a FFQ are presented in Table 2. Both NHW and Hispanic early adolescent boys had higher mean daily calcium intakes (mg) from all foods compared to their Asian counterparts. No significant differences in calcium intakes (mg) were seen among early adolescent girls. NHW boys and girls had significantly higher mean daily intakes of milk (cups/day) compared to their Hispanic counterparts. Hispanic girls had significantly higher mean soda intakes (cans/day) compared to Asian girls.

**Table 2:** Estimated dietary calcium intakes (mg/day) and patterns of milk and soda consumption among Asian, Hispanic and non-Hispanic white parents and their early adolescent children (aged 10–13 years) completing calcium-specific food frequency questionnaires

	*n*	Total		Asian		Hispanic		NHW	*P*-value ^1^
		Mean (SD)	*n*	Mean (SD)	*n*	Mean (SD)	*n*	Mean (SD)	
Parents ^2^	597		108		216		273		
Total calcium from food	∙∙∙	880 (453)	∙∙∙	693 (380) ^x^	∙∙∙	833 (433) ^y^	∙∙∙	991 (466) ^z^	< 0.001
Total dairy calcium intake	∙∙∙	720 (425)	∙∙∙	486 (354) ^x^	∙∙∙	682 (380) ^y^	∙∙∙	843 (440) ^z^	< 0.001
Milk as a beverage (cups/day)	∙∙∙	0.7 (0.9)	∙∙∙	0.5 (0.7) ^x^	∙∙∙	0.5 (0.6) ^x^	∙∙∙	0.9 (1.0) ^y^	< 0.001
Soda pop (cans/day)	∙∙∙	0.4 (0.6)	∙∙∙	0.3 (0.4) ^x^	∙∙∙	0.3 (0.5) ^x^	∙∙∙	0.6 (0.7) ^y^	< 0.001
Children ^3^									
Total calcium from food									
Total sample	597	1100 (504)	108	953 (464) ^x^	216	1098 (497) ^y^	273	1160 (514) ^y^	0.001
Boys	265	1170 (512) ^a^	51	957 (429) ^x^	91	1173 (514) ^y^	123	1256 (519) ^y, a^	0.002
Girls	332	1044 (492) ^b^	57	950 (497)	125	1043 (480)	150	1081 (499) ^b^	0.231
Total dairy calcium intake									
Total sample	597	962 (478)	108	801 (445) ^x^	216	947 (455) ^y^	273	1037 (493) ^y^	< 0.001
Boys	265	1033 (489) ^a^	51	807 (405) ^x^	91	1024 (473) ^x, a^	123	1133 (504) ^y, a^	< 0.001
Girls	332	905 (463) ^b^	57	796 (482)	125	890 (435) ^b^	150	958 (472) ^b^	0.071
Milk as a beverage (cups/day)									
Total sample	597	1.4 (1.2)	108	1.4 (1.2) ^x^	216	1.1 (1.0) ^y^	273	1.6 (1.3) ^x^	< 0.001
Boys	265	1.5 (1.2) ^a^	51	1.5 (1.2) ^x,y^	91	1.2 (1.0) ^x^	123	1.8 (1.3) ^y^	0.001
Girls	332	1.3 (1.2) ^b^	57	1.3 (1.1) ^x,y^	125	1.0 (1.0) ^x^	150	1.5 (1.2) ^y^	< 0.001
Soda pop (cans/day)									
Total sample	597	0.3 (0.5)	108	0.2 (0.3) ^x^	216	0.4 (0.5) ^y^	273	0.3 (0.5) ^x^	0.001
Boys	265	0.3 (0.5)	51	0.3 (0.4) ^a^	91	0.4 (0.6)	123	0.3 (0.4)	0.055
Girls	332	0.3 (0.5)	57	0.2 (0.2) ^x, b^	125	0.4 (0.5) ^y^	150	0.3 (0.5) ^x, y^	0.005

*NHW* non-Hispanic white, *SD* standard deviation

^1^*P*-values given represent differences between ethnic groups

^2^Values with different superscripts (x, y, z) indicate significantly different means between ethnic groups (i.e. across columns)

^3^Values with different superscripts (a, b) indicate significantly different means between boys and girls (i.e. within columns)

Sex-adjusted regression coefficients of calcium (mg), milk (cups/day), and soda (cans/day) intakes between parent-child pairs are shown in Table 3. Calcium intakes of parents and children showed significant positive associations among all ethnic groups. A significant correlation in frequency of soda consumption (cans/day) was found only among Hispanic parent-child dyads.

**Table 3:** Association between parents’ and children’s (aged 10–13 years) calcium, milk, and soda intakes: adjusted regression coefficients stratified by ethnicity^a^

	Total	Asian	Hispanic	NHW
	Parents vs. Children (*n* = 597 pairs)	Parents vs. Children (*n* = 108 pairs)	Parents vs. Children (*n* = 216 pairs)	Parents vs. Children (*n* = 273 pairs)
Total calcium from food (mg)	0.296***	0.275*	0.424***	0.164*
Total dairy calcium (mg)	0.307***	0.301*	0.386***	0.188**
Milk as a beverage (cups/day)	0.380***	0.309	0.317**	0.356***
Soda pop consumption (cans/day)	0.130***	0.124	0.343***	0.070

^a^Adjusted for sex of child

NHW = non-Hispanic white

* *P* < 0.05 ** *P* < 0.01 ***P < 0.001

### Linear regression modeling

#### Model 1

Model 1 (Table 4) included parents’ total calcium intake from all foods (as a marker for parental modeling), parent’s perceived intolerance to milk and dairy foods, home availability of CRF, parental rules and expectations for their child’s intake of beverages, and parent’s perception of convenience/ease of CRF. The interaction terms between parental rules and expectations and parents’ calcium intake with ethnic group were statistically significant. This model with the interaction terms explained 19% of the variance in the early adolescents’ calcium intakes.

**Table 4:** Multivariate linear regression for total dietary calcium from food (mg) of early adolescents (dependent variable) and parental psychosocial factors (independent variables)^ab^

Y = total dietary calcium (mg)						
	Model 1 ^c^			Model 2 ^d^		
	R^2^ = 0.190			R^2^ = 0.182		
	β ± SE	t-value	*P*-value	β ± SE	t-value	*P*-value
Parental psychosocial factors						
*Attitudes and Preferences*						
Perceived intolerance to dairy foods and milk	+ 57 ± 29	1.98	0.048	+ 50 ± 30	1.69	0.093
Convenience of CRF	+ 42 ± 37	1.143	0.253	+ 40 ± 38	1.07	0.287
Rules and expectations for child’s intake of beverages	+ 386 ± 61	6.31	< 0.001	+ 365 ± 65	5.58	< 0.001
Interaction terms						
NHW x rules and expectations	reference			reference		
Asian x rules and expectations	− 229 ± 117	−1.95	0.051	− 251 ± 126	−1.99	0.047
Hispanic x rules and expectations	−260 ± 93	−2.79	0.006	− 335 ± 99	−3.38	0.001
*Social and Environmental*						
Availability of CRF	+ 114 ± 41	2.79	0.006	+ 131 ± 41	3.20	0.001
Parental calcium intake/role modeling ^cd^	+ 0.11 ± 0.06	1.70	0.089	+ 85 ± 71	1.20	0.230
Interaction terms						
NHW x parent calcium intake/role modeling	reference			reference		
Asian x parent calcium intake/role modeling	+ 0.20 ± 0.14	1.50	0.135	+ 97 ± 143	0.68	0.500
Hispanic x parent calcium intake/role modeling	+ 0.23 ± 0.10	2.26	0.024	+ 257 ± 112	2.29	0.022

*SE* standard error, *NHW* non-Hispanic white

^a^Adjusted for ethnic group (indicator variable), gender, and parental employment status

^b^β represents difference in average calcium intakes (mg) among the children based on variables as labeled and adjusted for variables in the table

^c^Model 1 uses parent’s dietary calcium intake as estimated from the FFQ as a proxy for parental role modeling

^d^Model 2 uses the psychosocial factor, parental role modeling, as a proxy for parental dietary calcium intake

The beta-coefficient for parents’ perception of their intolerance to milk and dairy foods was statistically significant. The positive value would be interpreted as the adjusted difference in calcium intake (mg) by increment of the perceived intolerance scale [1–6]. A statistically significant positive association was also found for the availability of CRF in the home, parents’ calcium intake and parental rules and expectations, with children’s calcium intakes. When the interaction terms were added, parents’ calcium intake, although still positive, was no longer statistically significant for NHW or Asian children, but remained significant among the Hispanic children such that for each milligram greater calcium intake of the parent, calcium intake of the child would be 0.34 mg higher (0.11 mg plus 0.23 mg). However, for parental rules and expectations, the interaction term did not eliminate the significance of this scale from any of the ethnic groups. Rather, the strength of the relationship for rules and expectations by ethnic group was NHW > Asian>Hispanic.

#### Model 2

Model 2 (Table 4) includes the PSF role modeling as a proxy for parental dietary calcium intake and the variables described above in Model 1. As in Model 1, the interaction terms between parental rules and expectations and parental role modeling with ethnic group were statistically significant and performed the same as in Model 1 with regard to the association of the factors by ethnic group. In contrast to Model 1, parents’ perception of their intolerance to milk and dairy foods was not statistically significantly associated with the children’s calcium intakes. The “convenience” scale was retained in both models as its exclusion affected the significance of other PSF.

#### Additional models

Multivariate linear regression analyses were repeated with total dairy calcium intake of children as the dependent variable (Table 5). These models explained 21% of the variance in children’s dairy calcium intake. Findings were similar, with the exception of the subscale “intolerance,” which did not show a significant association with dairy calcium intake.

**Table 5:** Multivariate linear regression for total dairy calcium (mg) of early adolescents (dependent variable) and parental psychosocial factors (independent variables)^ab^

Y = total dairy calcium (mg)						
	Model 1 ^c^			Model 2 ^d^		
	R^2^ = 0.211			R^2^ = 0.206		
	β ± SE	t-value	*P*-value	β ± SE	t-value	*P*-value
Parental psychosocial factors						
Attitudes and Preferences						
Perceived intolerance to dairy foods and milk	+ 40 ± 27	1.50	0.140	+ 25 ± 27	0.91	0.361
Rules and expectations for child’s intake of beverages	+ 402 ± 58	6.99	< 0.001	+ 388 ± 61	6.35	< 0.001
Interaction terms						
NHW x rules and expectations	reference			reference		
Asian x rules and expectations	− 214 ± 109	−2.00	0.051	− 222 ± 117	−1.90	0.058
Hispanic x rules and expectations	−257 ± 87	−2.94	0.003	− 323 ± 92	− 3.52	< 0.001
Social and Environmental						
Availability of CRF	+ 129 ± 38	3.36	0.001	+ 148 ± 38	3.88	< 0.001
Parental dairy calcium intake/role modeling ^cd^	+ 0.10 ± 0.06	1.69	0.091	+ 66 ± 66	0.99	0.321
Interaction terms						
NHW x parent dairy calcium intake/role modeling	reference			reference		
Asian x parent dairy calcium intake/role modeling	+ 0.21 ± 0.14	1.51	0.132	+ 62 ± 134	0.47	0.64
Hispanic x parent dairy calcium intake/role modeling	+ 0.20 ± 0.10	1.99	0.048	+ 244 ± 102	2.39	0.017

*SE* standard error, *NHW* non-Hispanic white

^a^Adjusted for child ethnic group and gender, and parental employment status

^b^β represents difference in average dairy calcium intakes (mg) among the children based on variables as labeled and adjusted for variables in the table

^c^Model 1 uses parent’s dairy calcium intake as estimated from the FFQ as a proxy for parental role modeling

^d^Model 2 uses the psychosocial factor, parental role modeling, as a proxy for parental dietary calcium intake

## Discussion

Qualitative research has indicated parents potentially influence their children’s intake of CRF [15, 20, 22, 23]. The primary aim of this cross-sectional study involving Asian, Hispanic, and NHW parent-child pairs was to quantify the predictive value of parental calcium intakes and significant PSF, toward calcium intakes of their early adolescent children aged 10–13 years. In multivariate models, home availability of CRF, parental rules and expectations for their child’s intake of beverages, and parental role modeling overwhelmed all other PSF in predicting children’s calcium intakes. Under conditions of parents’ calcium intakes being associated with children’s total calcium intake, parental perceived milk and dairy intolerance was also important.

Several studies have suggested dietary behaviors of children are related to household availability of foods and beverages [24, 36]. Similarly, the availability of CRF in the home has been found to be positively associated with both boys’ [24, 37] and girls’ [37, 38] calcium intakes. The present study, from the parent’s perspective, suggests that home availability of CRF is one of the most important social and environmental factors influencing children’s calcium intakes, corroborating previous research findings from the child’s perspective [14]. Similarly, availability is considered one of the most important factors influencing children’s fruit and vegetable consumption [29]. This is the first study to demonstrate that availability is important among Asian, Hispanic, and NHW households using total availability of CRF in the home as opposed to availability being defined as milk served with meals [14, 24, 38]. In this study, milk being served with meals was assessed as a form of parental role modeling which was significantly associated with calcium intake among the Hispanic children only. Arcan et al. [39] demonstrated longitudinally that availability tracks with higher intakes of milk between high school and young adulthood. Therefore, creating an environment of constant access to CRF in the home can potentially start a lifetime of improved calcium intakes.

The significant interaction terms between parental rules and expectations with ethnic group suggests cultural differences in family interactions exist. This finding corroborates previous findings of factors influencing children’s fruit and vegetable [40, 41] and soft drink intake [42]. Results extend qualitative research findings suggesting differences between ethnic groups regarding parental rules and expectations for their child’s intake of beverages [15, 20]. Parental rules and expectations were positively associated with children’s total calcium intake from all foods and children’s total dairy calcium intake. This factor was important for all 3 ethnic groups but the influence of parental rules and expectations was by far greater among the NHW children than among the other groups.

A study by Hanson et al. [24] which was also based on parent report of the home food environment found no significant interaction between parents’ intake of dairy foods and multiple ethnic groups (i.e. White, Black, Asian, Hispanic, and other/mixed). Findings from the current study found parental modeling to be synonymous with parent’s calcium intake. With regard to the influence of parental role modeling on their children’s calcium intakes, findings suggest the family dynamics likely differ between Asian, Hispanic, and NHW households. Among Asians, cultural practices may dictate parental modeling of milk consumption is low, as milk is not part of the traditional Asian diet [19, 20]. Parental modeling of intake of CRF was significantly associated with children’s calcium intakes among Hispanic dyads only, after adjusting for multiple confounding factors in linear regression analysis. Parental modeling of intake has been identified as one of the most important psychosocial predictors of adolescents’ dietary choices [43, 44]. Findings from the current study suggest the influence of parental role modeling is greater among Hispanic children than among the NHW- and Asian children and this was also true when limited to dairy calcium sources.

In contrast to a priori hypothesis, parental perceived intolerance to milk and dairy foods was positively associated with their children’s total calcium intake from all foods in model 1 (Table 4). This association was significant across all ethnic groups. Studies have reported lower dairy and calcium intakes among children with perceived milk intolerance [14, 45, 46]. However, the influence of parental perceived dairy intolerance on their children’s calcium intakes has not previously been investigated. Parents are usually responsible for preparing family meals. Parents who perceive themselves to be intolerant to milk and dairy foods may avoid preparing meals containing these foods. This behavior would, therefore, be expected to negatively influence a child’s calcium intake since dairy foods are the primary source of calcium in a child’s diet [47]. Parental perceived intolerance did not show a significant association with children’s dairy calcium intake when the model was repeated with total dairy calcium as the dependent variable (Table 5). Thus, parental perceived dairy intolerance is positively associated with children’s total calcium intake from all foods but is not associated with children’s total calcium from dairy foods, suggesting such parents promote the consumption of calcium-rich non-dairy foods for their children. Qualitative research has indicated that parents who did not consume dairy foods substituted these foods with calcium-supplements, soy milk, or other CRF, thereby continuing to act as role models positively influencing their children’s calcium intake [15].

Other PSF examined in this study did not show a significant association with children’s calcium intakes in multivariate analyses. This finding aligns with previous literature. Previous research has not revealed an association between parental knowledge of nutrition and calcium intakes of children across varying ages [13, 48]. The finding that parental concern about cost of CRF is not significantly associated with children’s calcium intake has also previously been noted [49]. Boutelle et al. [50] reported maternal report of concern regarding the healthfulness of their own diet was not significantly associated with dietary choices of their adolescent children. Similarly, parental concern over the adequacy of their own calcium intake was not identified as a salient factor associated with their children’s calcium intakes.

The multivariate models described in this paper accounted for 19–21% of the variance in children’s calcium intakes. Other studies have generally reported variances between 4 and 10% for explaining intakes of fruits and vegetables [26, 51]. Larson et al. [14] described a model explaining 71–72% of the variance in calcium intake among 11–18 years old adolescents using self-reported information from the adolescents. The majority of the variance (53–58%) was explained by energy intake alone. Omitting the contribution of energy, the model from Larson and colleagues [14] accounted for 13–19% of the variance. In the present study, energy was not considered a factor due to calcium requirements not being energy dependent [52]. Therefore, the influence of parents on the calcium intakes of their early adolescent children may be greater than previously presumed. Recommendations for effective interventions have highlighted the importance of addressing mediating variables with strong relationships to dietary intake [27]. This may indicate the psychosocial variables in the models presented here may be more effective in promoting change in children’s dietary behavior, and certainly combined with previously reported children’s factors would be even more so.

Parent report of the home food environment was used in this study as it is generally believed that parents provide more objective information on their own dietary intake (role modeling) and their children’s accessibility to certain foods in the home [53]. However, perceptions concerning the home food environment can differ between parents and children [53] and it remains unclear whether parent or child report is more reliable [41]. Child-report of their parents’ behavior may be less biased than parents’ self-report of their own behavior [54]. As parental role modeling and parents’ calcium intakes were highly correlated and produced similar results when interchanged in multivariate models, social desirability bias was likely low. In addition, the use of a questionnaire for this purpose was practical but not ideal, as objective environmental measures are preferable to more thoroughly understand the association between parents and the home food environment, and children’s dietary behaviors [55].

A secondary aim of this study was to investigate associations in milk, soda, and calcium intakes among parent-child pairs. Several studies have suggested a resemblance in dietary patterns, such as reported intake of snacks [56], fruit and vegetables [42, 53], and dairy foods [24], among parent-child pairs. A positive association in frequency of milk consumption has been reported among mothers and their children aged 4–6 years [16] and aged 5–9 years [38]. Results from the current study involving adolescents aged 10–13 years, support these findings and suggest the association in milk consumption between parent-child pairs persists through early adolescence. In contrast to previous studies [18, 38], this study included cross-cultural comparisons in its analyses. Results suggest there is no significant association in milk consumption among Asian parent-child pairs (*P* = 0.07). The sample size of Asian dyads (*n* = 108) may have been too small as a sample of ≥180 pairs was identified as the desired sample size to detect a significant association. Studies have reported a positive association in intake of carbonated beverages among parent-child pairs [18, 42]. In the current study, a significant association in frequency of soda consumption was found among Hispanic parent-child dyads only, after adjusting for sex of child. This, together with findings from multivariate analysis, may suggest parental modeling of beverage intake may be more influential among Hispanic children. At least one other study has reported a stronger resemblance in soda consumption among Hispanic parent-child dyads compared to NHW dyads [42].

The large sample size strengthens the findings of this study. However, as convenience sampling was employed to recruit only Asian, Hispanic, and NHW participants, findings cannot be generalized to other ethnic groups or to the general population. A comparison of education level of parents in this study sample (59% Asians, 9% Hispanics, 52% NHWs with a 4 year college or advanced degree) to American adults > 25 years in the general population (55% Asians, 29% Hispanics 38% NHWs with a 4 year college or advanced degree) [57] indicates some sampling may have occurred in areas near universities which in turn may have resulted in a study sample including NHWs and Asians who were slightly more educated than the general U.S population for these groups. This may, in part, explain the low percentage of individuals with implausible calcium intakes [58]. As the distribution of ethnic groups varied by state due to clustering of specific ethnic groups in different parts of the U.S, it is not possible to report with certainty that differences reported are due to ethnic- rather than geographical differences, despite adjusting for state in multivariate analyses. Due to the cross-sectional nature of this study’s design, no causal relationships can be assumed. In addition, data were collected in 2006–2008, and parental influences on calcium intakes may have changed since then. A major strength of this study was the use of the same FFQ to assess calcium intakes of children and their parents. However, FFQ’s can only provide estimates of nutrients intakes which are not comparable to actual intakes. Further, this tool did not allow for examination of the distribution of calcium intake throughout the day to determine when most CRF were consumed. Also of note, lactose maldigestion testing was not undertaken among the parents and their children in this study [45]. Avenues to increase calcium intake in lactose maldigesters need further exploration in subsequent studies [5]. Finally, this study did not assess how calcium rich foods such as milk were made available regarding purchasing, such as whether consumption increased when dairy products were on sale.

In respect to the limitations identified regarding the study design, longitudinal studies would aid with verifying causal relationships. One longitudinal study provided evidence that availability of CRF in the home tracks with higher dairy intakes from late adolescence to young adulthood but failed to demonstrate an association from early to late adolescence [39]. Similar to these findings, the current study suggests other household factors, in addition to availability, are also important. As models explained 19–21% of the variance in children’s calcium intakes, findings support previous research [55], i.e. that the home environment represents only one component of the broader environment in which early adolescents live. Future studies should quantify the variance in children’s calcium intakes explained by other important environmental factors, such as peers and school settings.

### Conclusions, applications and implications for further research

In the current study, parental modeling of intake, parental rules and expectations for the consumption of CRF, and home availability of CRF, were identified as the most important parental PSF in predictive models of children’s total and dairy calcium intakes. In previous studies, adolescents have reported low levels of parental role modeling and limited rules and expectations for their consumption of CRF [19, 20]. In one study, only one third of adolescents reported that CRF were available in their home [20]. Therefore, future intervention programs should be directed at increasing parents’ awareness of how their attitudes and behaviors may influence calcium intakes of their children. Findings also suggest that ethnic background may be an important factor to consider when designing intervention programs. Among Hispanic families, interventions which focus on increasing parental modeling of intake of CRF, and decreasing parental intake of carbonated beverages, may be most successful.

In conclusion, results from this study provide insight into the degree to which parents can improve calcium intake of their early adolescent children. Findings can help guide the development of effective nutrition interventions aimed at improving calcium intakes of children of ethnic backgrounds at highest risk for osteoporosis during a critical time of bone accretion. Findings suggest interventions to improve children’s calcium intakes should also focus on parents and in particular provide guidance on improving home availability of CRF, role modeling, and developing rules and expectation for the consumption of such foods. In addition, recognition that these family interactions vary between Asian, Hispanic, and NHW households may enhance the effectiveness of interventions.

## Additional files

Additional file 1: Parent psychosocial questionnaire; questionnaire evaluating parental PSF hypothesized to influence children’s calcium intakes. (PDF 40 kb)

Additional file 2: Flowchart of sample; flow diagram showing details of final sample size. (PDF 190 kb)

## Abbreviations

CRF: Calcium-rich foods
EFNEP: Expanded Food and Nutrition Education Program
FFQ: Food frequency questionnaire
NHW: Non-Hispanic white
PSF: Parental psychosocial factors
SCT: Social Cognitive Theory
SES: Socio-economic status
SNAP-Ed/formerly Food Stamp Nutrition Education Program: Supplemental Nutrition Assistance Program Education
USDA: United States Department of Agriculture
WIC: Special Supplemental Nutrition Program for Women, Infants, and Children
