# Supplementary material for: Parent and household influences on calcium intake among early adolescents
Source: BMC Public Health. 2018 Dec 19;18:1390. doi: 10.1186/s12889-018-6297-5 (PMC6300005; doi:10.1186/s12889-018-6297-5)
Supplement: Supplementary file 1 — Parent psychosocial questionnaire; questionnaire evaluating parental PSF hypothesized to influence children’s calcium intakes. (PDF 40 kb) [file 12889_2018_6297_MOESM1_ESM.pdf]

# Appendix

| |
|---|
| Social and environmental construct subscales with accompanying statements |
| Culture/tradition for parent (5-point scale; strongly disagree to strongly agree plus don't know) |
| I grew up eating/drinking cheese. |
| I grew up eating/drinking yogurt. |
| I grew up eating/drinking milk. |
| I grew up eating/drinking pizza. |
| I grew up eating/drinking dark green leafy vegetables. |
| Parent's perception of child's access to calcium rich foods outside of home (4-point scale; Strongly disagree to strongly agree plus don't know) |
| My child gets calcium in foods when served at school. |
| My child gets calcium from milk when served at school. |
| Parenting styles (5-point scale; strongly disagree to strongly agree plus don't know) |
| Even if my child doesn't like what is fixed for meals, he/she still has to eat at least some of it. |
| I help my child decide what to eat or drink. |
| My child is encouraged to eat healthy foods when he/she sees me eating healthy foods. |
| By making healthy foods available at home, I encourage my child to eat healthy. |
| Parental role modeling (5-point scale; never to always plus don't know) |
| How often do YOU do the following? |
| Eat foods high in calcium. |
| Drink regular soda pop. (Reverse code) |
| Drink diet soda pop. (Reverse code) |
| Drink milk with breakfast. |
| Drink milk with lunch. |
| Drink milk with dinner. |
| Drink milk with snacks. |
| Drink water with meals. (Reverse code) |
| Drink water with snacks. (Reverse code) |
| Drink tea with meals. (Reverse code) |
| Drink tea with snacks. (Reverse code) |
| Drink milk with meals away from home. |
| Eat yogurt with meals. |
| Eat yogurt with snacks. |
| Availability of calcium-rich foods (5-point scale; never to always) |
| How often do you keep these foods in your home for YOUR CHILD? |
| Cheese |
| Yogurt |
| Cereal and milk |
| Macaroni and cheese |
| Milk |
| Chocolate milk |
| Juice with added calcium |
| How often do you have these beverages in your home? (Reverse code) |
| Regular soda pop |
| Diet soda pop |
| Fruit drinks like Kool-Aid® or Sunny Delight® |
| Parent's concerns about prices of calcium-rich foods (5-point scale; strongly disagree to strongly agree plus don't know) |
| Cheese is expensive. |
| Yogurt is expensive. |
| Milk is expensive. |
| Pizza is expensive. |
| Dark green leafy vegetables are expensive. |
| Parental encouragement (5-point scale; strongly disagree to strongly agree plus don't know) |
| I want my child to eat healthy now so he/she will have good eating habits when he/she is older. |

(*Continued*)

Downloaded by [Weill Cornell Medical College] at 08:33 12 February 2016

(Continued).

| |
|---|
| I think it is important to talk to my child about eating healthy. |
| I tell my child that dairy foods are good for him/her. |
| My child is encouraged to eat healthy foods when he/she sees me eating healthy foods. |
| By making healthy foods available at home, I encourage my child to eat healthy. |
| Eating out (5-point scale; never to always) |
| We are too busy to cook. |
| We are too tired to cook. |
| We are taking children to activities at mealtime. |
| To spend quality time together as a family. |
| To celebrate (wedding, birthday) or as a reward (e.g., for good behavior). |
| To be with friends in a social setting. |
| To try something new. |
| Interruption of family meals (5-point scale; never to always) |
| Our family schedules are too busy for us to eat together. |
| Our family eats meals together in from of the TV. |
| At dinner everyone in our family is going in different directions, grabbing and running to eat on their own. |
| Attitude and preferences construct subscales with accompanying statements |
| Liking/taste calcium-rich foods for parent (6-point scale; strongly disagree to strongly agree plus don't know) |
| I like the taste of cheese. |
| I like the taste of yogurt. |
| I like the taste of milk. |
| Dark green leafy vegetables (like collard greens, kale and bok choy) taste good. |
| I don't know how to fix dark green leafy vegetables so my family will like them. (Reverse code) |
| I like the taste of tofu. |
| I don't know how to fix tofu so my family will like them. (Reverse code) |
| Dairy/milk intolerance for parent (6-point scale; strongly disagree to strongly agree plus don't know) |
| Yogurt upsets my stomach. |
| Milk makes me sick. |
| I heat milk to make it easier to digest. |
| I eat cheese or yogurt because milk upsets my stomach. |
| Health benefits for parent (6-point scale; strongly disagree to strongly agree plus don't know) |
| Cheese gives me calcium. |
| Cheese is good for me. |
| Yogurt is good for me. |
| Milk is good for me. |
| Milk gives me calcium. |
| Milk is a good source of protein. |
| Pizza is good for me. |
| Pizza is healthy, if you have healthy toppings. |
| Dark green leafy vegetables are good for me. |
| Tofu is good for me. |
| Parent's perception of importance of calcium sources for children (5-point scale; not at all important to extremely important) |
| In your opinion, how important are these foods in helping YOUR CHILD get the calcium he/she needs? |
| Macaroni and cheese |
| Pizza |
| Milk |
| Yogurt |
| Cheese |
| Dark green leafy vegetables |
| Tofu |
| Weight concerns in general (6-point scale; strongly disagree to strongly agree plus don't know) |
| Cheese is fattening. |
| Milk is fattening. |

(*Continued*)

Downloaded by [Weill Cornell Medical College] at 08:33 12 February 2016

(Continued).

| |
|---|
| Pizza is fattening. |
| I think lower fat dairy foods are better for weight control than higher fat dairy foods. |
| Parent's expectations for child's intake of beverages (6-point scale; strongly disagree to strongly agree plus don't know) |
| How often do you allow YOUR CHILD to drink these beverages in your home? |
| Regular soda pop. (Reverse code) |
| Diet soda pop. (Reverse code) |
| Fruit drinks like Kool-Aid® or Sunny Delight®. (Reverse code) |
| How often do you try to get YOUR CHILD to do the following? |
| Drink milk with breakfast. |
| Drink milk with snacks. |
| Drink milk with lunch. |
| Drink milk with dinner. |
| Drink milk when we go out. |
| Drink water with meals. (Reverse code) |
| Drink water with snacks. (Reverse code) |
| Drink tea with meals. (Reverse code) |
| Drink tea with snacks. (Reverse code) |
| Drink less soda pop. |
| Parent's concern over adequacy of own calcium intake (6-point scale; strongly disagree to strongly agree plus don't know) |
| If I don't get enough calcium, I could have weak bones as I get older. |
| I am concerned that I am not getting enough calcium. |
| I am not getting enough calcium because I don't eat dairy products. |

Downloaded by [Weill Cornell Medical College] at 08:33 12 February 2016
