# Supplementary material for: Parent and household influences on calcium intake among early adolescents
Source: BMC Public Health. 2018 Dec 19;18:1390. doi: 10.1186/s12889-018-6297-5 (PMC6300005; doi:10.1186/s12889-018-6297-5)
Supplement: Supplementary file 2 — Flowchart of sample; flow diagram showing details of final sample size. (PDF 190 kb) [file 12889_2018_6297_MOESM2_ESM.pdf]

Flow diagram showing details of final sample size

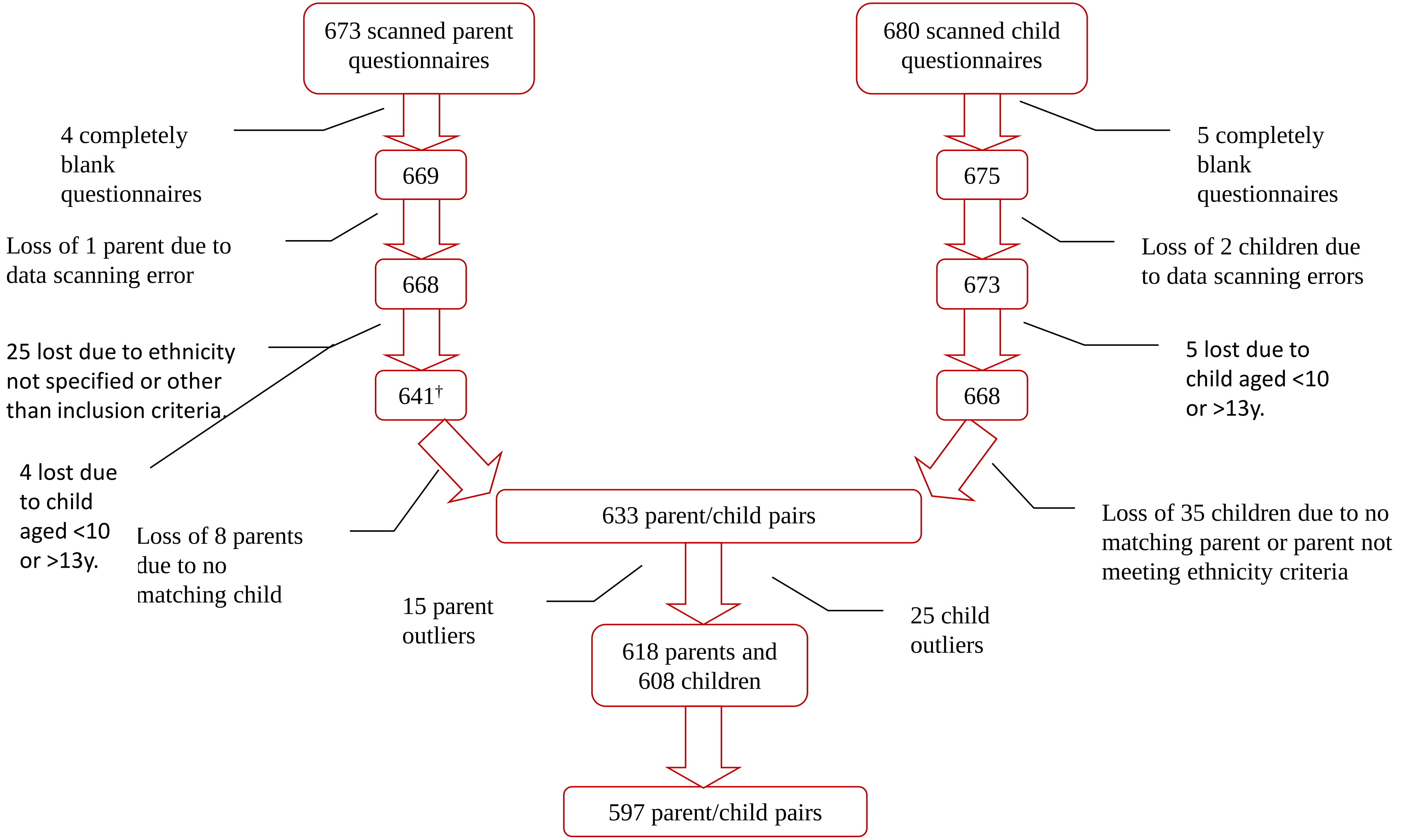

†In some cases, participants did not meet inclusion criteria for more than one reason, therefore duplicates exist and numbers do not add up.
